# Dietary AGEs involvement in colonic inflammation and cancer: insights from an *in vitro* enterocyte model

**DOI:** 10.1038/s41598-020-59623-x

**Published:** 2020-02-17

**Authors:** Ovidiu I. Geicu, Loredana Stanca, Sorina N. Voicu, Anca Dinischiotu, Liviu Bilteanu, Andreea I. Serban, Valentin Calu

**Affiliations:** 1 Department of Biochemistry and Molecular Biology, Faculty of Biology, University of Bucharest, 91-95 Blvd. Splaiul Independentei, 050095 Bucharest, Romania; 2 Department of Preclinic Sciences, Faculty of Veterinary Medicine, University of Agronomic Sciences and Veterinary Medicine of Bucharest, 105 Blvd. Splaiul Independentei, 050097 Bucharest, Romania; 3 Department of General Surgery, University of Medicine and Pharmacy “Carol Davila” Bucharest, 8 Blvd., Eroii Sanitari, 050474 Bucharest, Romania

**Keywords:** Interleukins, Cancer models

## Abstract

The number of colon cancer cases is increasing worldwide, and type II diabetes patients have an increased risk of developing colon cancer. Diet-borne advanced glycation end-products (AGEs) may promote neoplastic transformation; however, the mechanisms involved remain elusive. The present study helped to define the relationship between dietary AGEs and cancer progression. C2BBe1 adenocarcinoma enterocytes were exposed to 200 µg/mL glycated casein (AGEs-Csn) for up to 24 h. AGEs-Csn exposure resulted in increased cell proliferation, maladaptative changes in SOD and CAT activity and moderate levels of hydrogen peroxide (H_2_O_2_) intracellular accumulation. AGEs-Csn activated pro-survival and proliferation signalling, such as the phosphorylation of mTOR (Ser2448) and Akt (Ser473). GSK-3β phosphorylation also increased, potentially inducing extracellular matrix remodelling and thus enabling metastasis. Moreover, AGEs-Csn induced MMP-1, -3, -7, -9 and -10 expression and activated MMP-2 and MMP-9, which are regulators of the extracellular matrix and cytokine functions. AGEs-Csn induced inflammatory responses that included extracellular IL-1β at 6 h; time-dependent increases in IL-8; RAGE and NF-κB p65 upregulation; and IκB inhibition. Co-treatment with anti-RAGE or anti-TNF-α blocking antibodies and AGEs-Csn partially counteracted these changes; however, IL-8, MMP-1 and -10 expression and MMP-9 activation were difficult to prevent. AGEs-Csn perpetuated signalling that led to cell proliferation and matrix remodelling, strengthening the link between AGEs and colorectal cancer aggressiveness.

## Introduction

Worldwide, the annual cumulative number of deaths related to diabetes and colon cancer that were recorded in the last 20 years has increased by 90%, while in developed countries, it rose by 57%. The risk of developing colon cancer was estimated to be 27% greater in patients with type II diabetes than in those not afflicted by this disease^1^. This association is thought to be strengthened by lifestyle choices such as a diet rich in carbohydrates and advanced glycation end-products (AGEs) compounds; however, the molecular mechanisms responsible for this association remain elusive. A growing body of data has indicated that colon cancer and type II diabetes may involve shared signalling pathways, such as the activation of the inflammatory response via NF-κB, the activation of the Wnt/β-catenin pathway, iron homeostasis imbalances and increased oxidative stress^2,3^. Research has also shown that tumours tolerated higher levels of ROS than healthy tissue, most likely due to increased basal activity of antioxidative enzymes^4^. Surprisingly, this state may also be linked to increased cell proliferation and differentiation^5^ and even to increased invasiveness and drug tolerance in certain tumours^6^.

In cancerous cells, RAGE, which is sometimes referred to as the tumour receptor, is typically overexpressed^7–9^. The perpetuation of signalling involving the AGEs ligands, their receptor RAGE and the NF-κB transcription factor has recently been shown to form a positive feedback loop^10,11^, which is critical in a wide spectrum of afflictions and their complications^12,13^. The NF-κB transcription factor regulates the transcription of a wide array of genes involved in inflammation, immune cell development, cell cycle progression and cellular proliferation and death, thus representing a key player in cancer, diabetes, and autoimmune, inflammatory, and neurodegenerative diseases^14–16^. Numerous studies have pointed out that there is a close link between the activation of the PI3K/Akt pathway and the development of various types of cancers^17^, and preliminary results showed that Akt phosphorylation is rapidly induced *in vitro* following AGEs exposure^18^. A more comprehensive understanding of the molecular mechanisms that strengthen these associations would be clinically relevant and would help to improve treatment plans. The present study aimed to advance the knowledge of the relationship between cancerous enterocyte responses to AGEs exposure and to clarify the link between high dietary AGEs intake and cancer evolution by describing the molecular pathways that are modulated. Thus, we performed an *in vitro* study with human cancer cells with an enterocyte morphology that were treated with glycated casein (AGEs-Csn) for 3, 6, 9 and 24 h and with the specific blocking antibodies anti-RAGE, anti-TNF-α or anti-IL-1β.

## Results and Discussion

### Cell proliferation and viability of C2BBe1 cells during AGEs-Csn treatment

**Figure 1:**
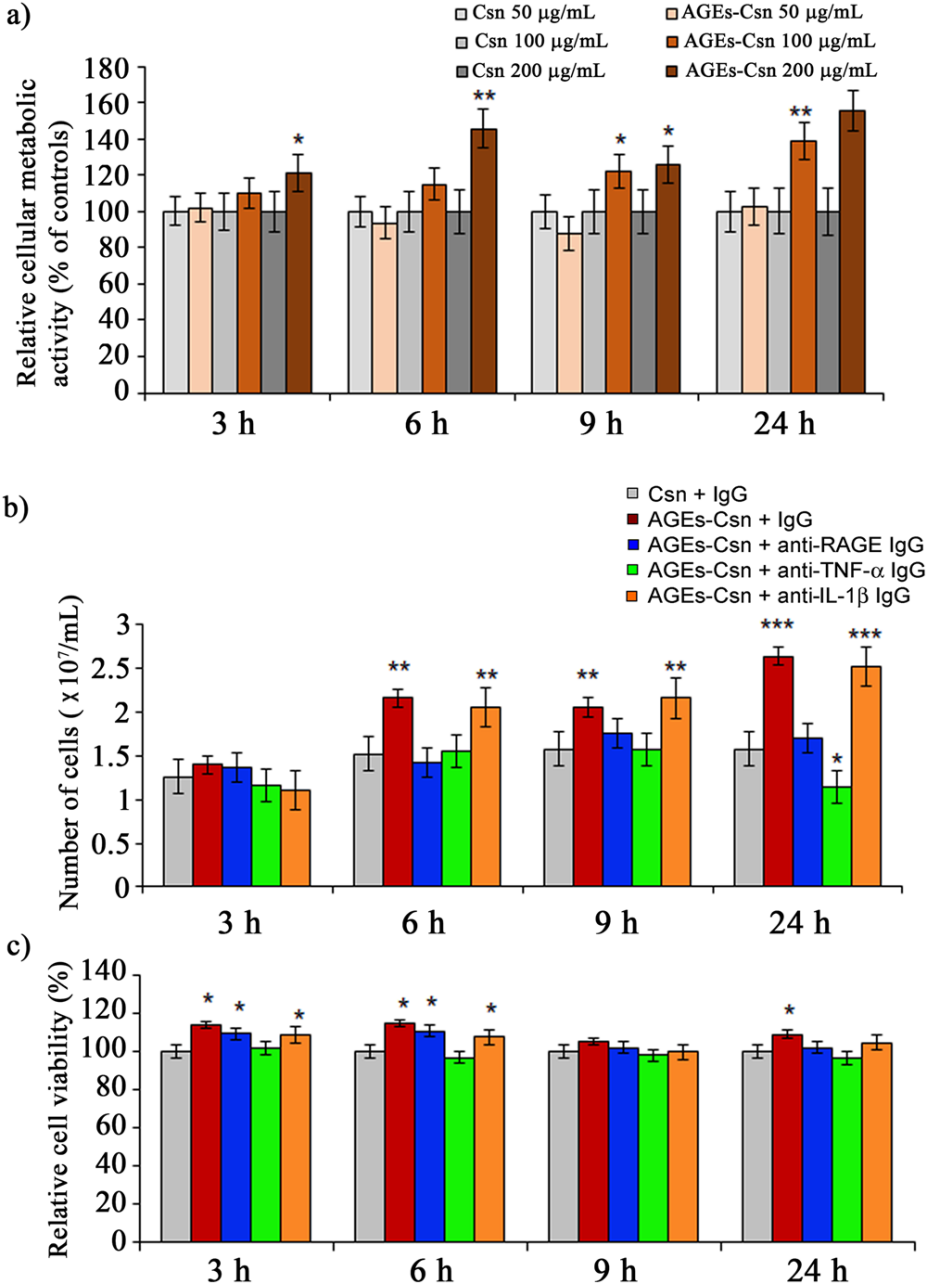
The metabolic activity, proliferation and viability of AGEs-exposed C2BBe1 cells. (**a**) The relative metabolic activity of cells exposed to 50, 100 or 200 µg/mL AGEs-Csn, as assessed by the MTT assay. (**b**) The absolute cell numbers and (**c**) the cellular viability after treatment with 200 µg/mL AGEs-Csn and blocking antibodies. Live and dead cells were counted (dye exclusion assay), and viability was calculated according to the formula given in the methods section. The data represent the means of three independent experiments. Statistical analysis was performed on data groups used to calculate the means. Student’s t-test were performed using the Quattro Pro X3 software (Version 13.0.0.406, Mountain View, CA, USA), and statistically significant changes (compared to the control at the relevant time-point) were noted as *p ≤ 0.05; **p ≤ 0.01; and ***p ≤ 0.001.

Three different doses of AGEs-Csn or non-glycated Csn (50, 100 and 200 µg/mL) were used to treat C2BBe1 cells for 3, 6, 9 or 24 h. A 3-(4,5-dimethylthiazol-2-yl)-2,5-diphenyltetrazolium bromide (MTT) test revealed that the metabolic activity of the cells increased in response to treatment with AGE-Csn. After 3 h, the metabolic activity had increased by 21% in cells exposed to 200 µg/mL AGEs-Csn. After 6 h, the cellular metabolic activity also increased in the cells treated with a 100 µg/mL dose, and after 24 h, all the AGEs-Csn doses resulted in increased cell metabolic activity, reaching 102%, 139% and 155% of the control levels, respectively (Fig. 1a). Based on these data, and considering the literature reports of a daily dietary AGEs intake of 25 to 75 mg AGEs^19^, and that the estimated surface area of the human colon is approximately 2 m^2^ ^20^, we selected a dose of 200 µg/mL AGEs-Csn for further experiments. This dose was just over the upper limit of the normal range and simulated a diet rich in carbohydrates and AGEs compounds. To identify potential molecular mechanisms that could explain this increase in metabolic activity, we also treated AGEs-Csn-exposed cells with the blocking antibodies anti-RAGE, anti-TNF-α or anti-IL-1β, and non-immunogenic IgG was used as a control. After 6 h of treatment, an increase in cell proliferation was noted for the cells that were co-treated with 200 µg/mL AGEs-Csn and non-immunogenic IgG or an anti-IL-1β antibody, as the cell counts increased by 0.64 × 10^7^ cells/mL and 0.54 × 10^7^ cells/mL, respectively, compared to the control cell counts (Fig. 1b). Another proliferation increase was detected after 24 h in both conditions, when the number of cells exceeded 2.5 × 10^7^ cells/mL, while the control cells number was 1.58 × 10^7^ cells/mL. The anti-RAGE and anti-TNF-α blocking antibodies maintained cell proliferation at the control levels for up to 9 h of AGEs-Csn exposure; however, at the last 24-h interval, the anti-TNF-α antibody co-treatment surprisingly diminished the cell numbers by 0.44 × 10^7^ cells/mL compared to the controls (Fig. 1b). In a study conducted on 1321N1 glioblastoma cells, TNF-α stimulated cell proliferation via an Akt phosphorylation-dependent mechanism that involved the activation of cyclin D expression^21^. A similar mechanism could contribute to the decrease in cell proliferation that was induced in our study by anti-TNF-α antibodies.

On the one hand, in cells co-treated with AGEs-Csn and non-immunogenic IgG, cell viability was maintained at slightly higher levels than in the control cells (Fig. 1c). Similar increases were observed at 3 and 6 h of AGEs-Csn and anti-RAGE or anti-IL-1β antibody co-treatments. On the other hand, the proliferation of colorectal carcinoma cells exposed to AGEs-Csn increased by up to 70% after 24 h (Fig. 1b). Similar patterns, in which AGEs had a more pronounced effect on cell proliferation than on cell viability, have been reported in breast cancer cells. Additionally, that study revealed that AGEs induced cell proliferation in a dose-dependent manner only up to the 100 µg/mL dose^22^; however, the exact concentration threshold may vary with the cellular phenotypes.

### AGEs-Csn treatment is associated with moderate oxidative imbalances and inflammation in C2BBe1 cells

The oxidative status of C2BBe1 cells exposed to AGEs-Csn and blocking antibodies was assessed by SOD and CAT enzyme activities and the intracellular hydrogen peroxide (H_2_O_2_) level as well as the total intracellular antioxidative capacity by the ferric reducing antioxidant power (FRAP) assay. As a measure of oxidative stress the carbonylated proteins were assessed. Our data indicated that there was a relatively high basal specific activity of SOD (on average 0.4036 U/mg of total protein) in non-glycated Csn-exposed cells. Notably, several previous studies have reported augmented MnSOD activity in malignant gastric tumours^23–25^, and it was shown that such cases were associated with lower patient survival rates^26^. Changes in the cellular redox status and inflammatory response are also common in colorectal cancers, with molecular outcomes that result in tumour progression^27^.

**Figure 2:**
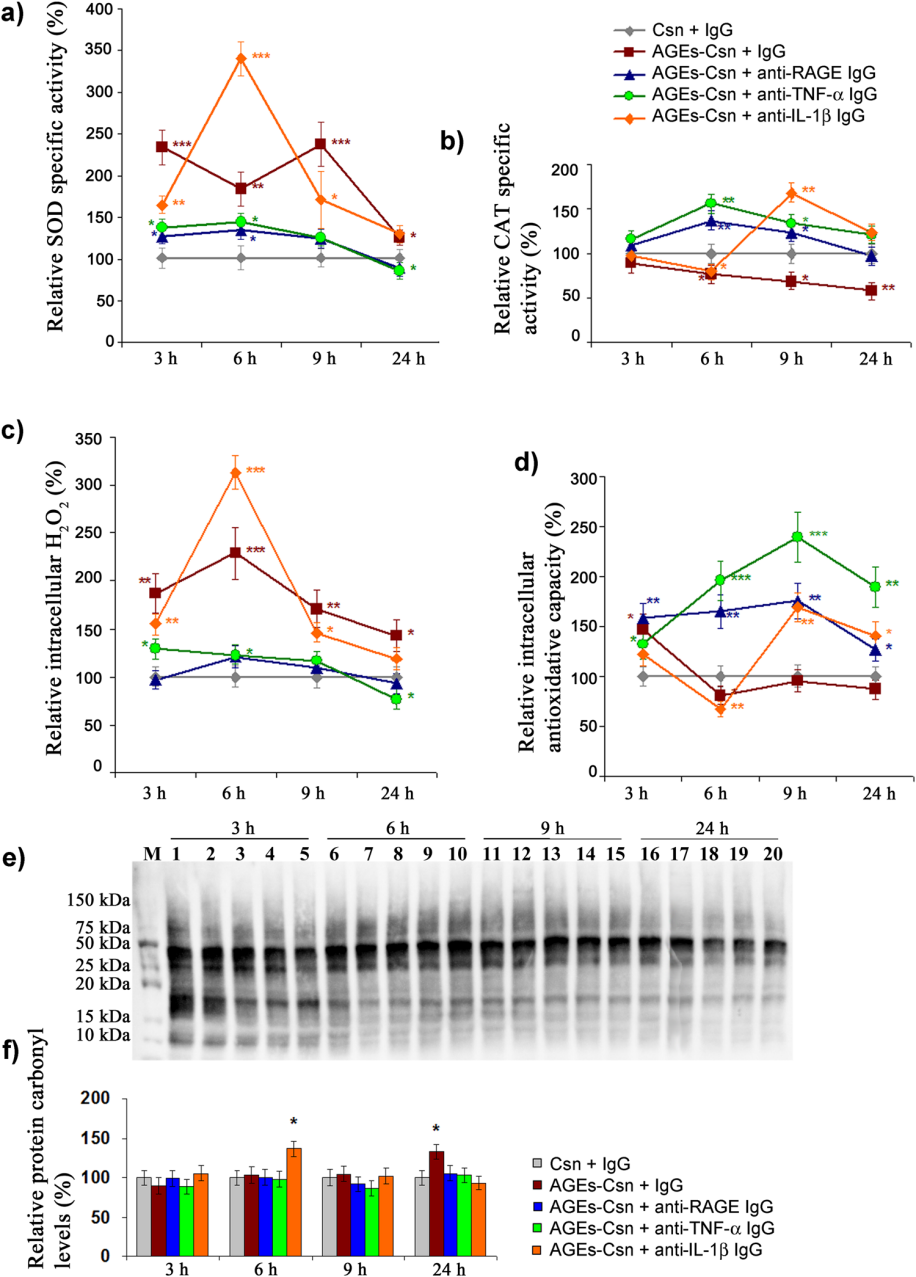
Oxidative status modulation following AGEs exposure and the effects of co-treatments with AGEs and anti-RAGE, anti-TNF-α or anti-IL1-β. (**a**) Relative intracellular total SOD specific activity levels. (**b**) Relative intracellular CAT specific activity levels. (**c**) Relative intracellular H_2_O_2_ levels. (**d**) Relative total intracellular antioxidative capacity. (**e**) Representative Western blot membrane stained for carbonylated proteins. Line M – molecular weight ladder, lines 1 to 5 correspond to samples at the 3-h time-point, as follows: 1 − Csn + non-immune IgG (control); 2 – 200 µg/mL AGEs + non-immune IgG; 3 – 200 µg/mL AGEs + anti-RAGE antibody; 4 – 200 µg/mL AGEs + anti-TNF-α antibody; and 5 – 200 µg/mL AGEs + anti-IL1-β antibody. Samples were loaded in the same order for all the time-points, as indicated above the membrane. The membrane was cropped to minimize the areas with no chemiluminescent signal (full-length blot is available in Supplementary Fig. 1). (**f**) Densitometry analysis data were normalized to the total loaded protein. Graph shows the relative level of carbonylated proteins in each condition relative to the control at that time-point. Statistical significance is noted as *p ≤ 0.05; **p ≤ 0.01; and ***p ≤ 0.001.

AGEs-Csn and non-immune IgG co-treatment induced a significant increase in SOD activity throughout the experimental interval, with levels that were 132%, 82%, 136% and 25% higher than controls after 3 h, 6 h, 9 h and 24 h, respectively. It should be noted, however, that the final 24-h time-point demonstrated the smallest relative increase in SOD activity. AGEs-Csn co-treatment with anti-RAGE or anti-TNF-α consistently reduced the SOD activity; surprisingly, these treatments reduced the SOD activity to below the control levels after 24 h (88% and 85% of the controls, respectively). AGEs-Csn and anti-IL-1β co-treatment had a rather unexpected effect. Although the IL-1β blocking antibody hindered the activation of SOD at all time-points except for one, after 6 h of exposure, there was a dramatic increase in SOD activity, which reached 338% of the control SOD activity (Fig. 2a). A recent paper demonstrated that certain types of monoclonal IL-1β antibodies have the potential to behave in an agonistic fashion by protecting the active sites of the target cytokine and prolonging their half-life^28^; this could provide some insight into this surprising result, which was not limited to the SOD activity, as we shall discuss further.

The transcriptional activation of *SOD* genes by oxidative stress as well as by pro-inflammatory cytokines, such as TNF-α and IL-1β, is well known, and the NF-κB family of transcription factors are known to be critical mediators of these processes^29^. Our results revealed that NF-κB p65 levels were the highest with AGEs-Csn and non-immunogenic IgG and AGEs-Csn and anti-IL-1β co-treatments, while NF-κB p50 expression was completely inhibited at the 6-h time-point; the protein expression of NF-κB family of transcription factors will be further discussed in the following paragraphs. Interestingly, the overexpression of p50 was shown to inhibit *MnSOD* gene transcription that was activated by cytokines^30^.

Contrary to the activation pattern reported for SOD, AGEs-Csn and non-immunogenic IgG co-treatment inhibited CAT activity at all the time-points studied, with the largest decrease (40% compared to the basal level) recorded at the 24-h time-point (Fig. 2b). Most significantly, anti-RAGE and anti-TNF-α induced CAT activity, especially at the 6-h and 9-h time-points. AGEs-Csn and anti-IL-1β co-treatment induced an interesting modulation of CAT activity levels. Until 6 h of treatment, no significant changes were seen compared to the AGEs-Csn and non-immunogenic IgG conditions. However, at 9 h of exposure, the CAT activity significantly increased by 68% compared to the basal level and then receded at the 24-h time-point, although the CAT activity level remained higher than the level in the control (Fig. 2b).

**Figure 3:**
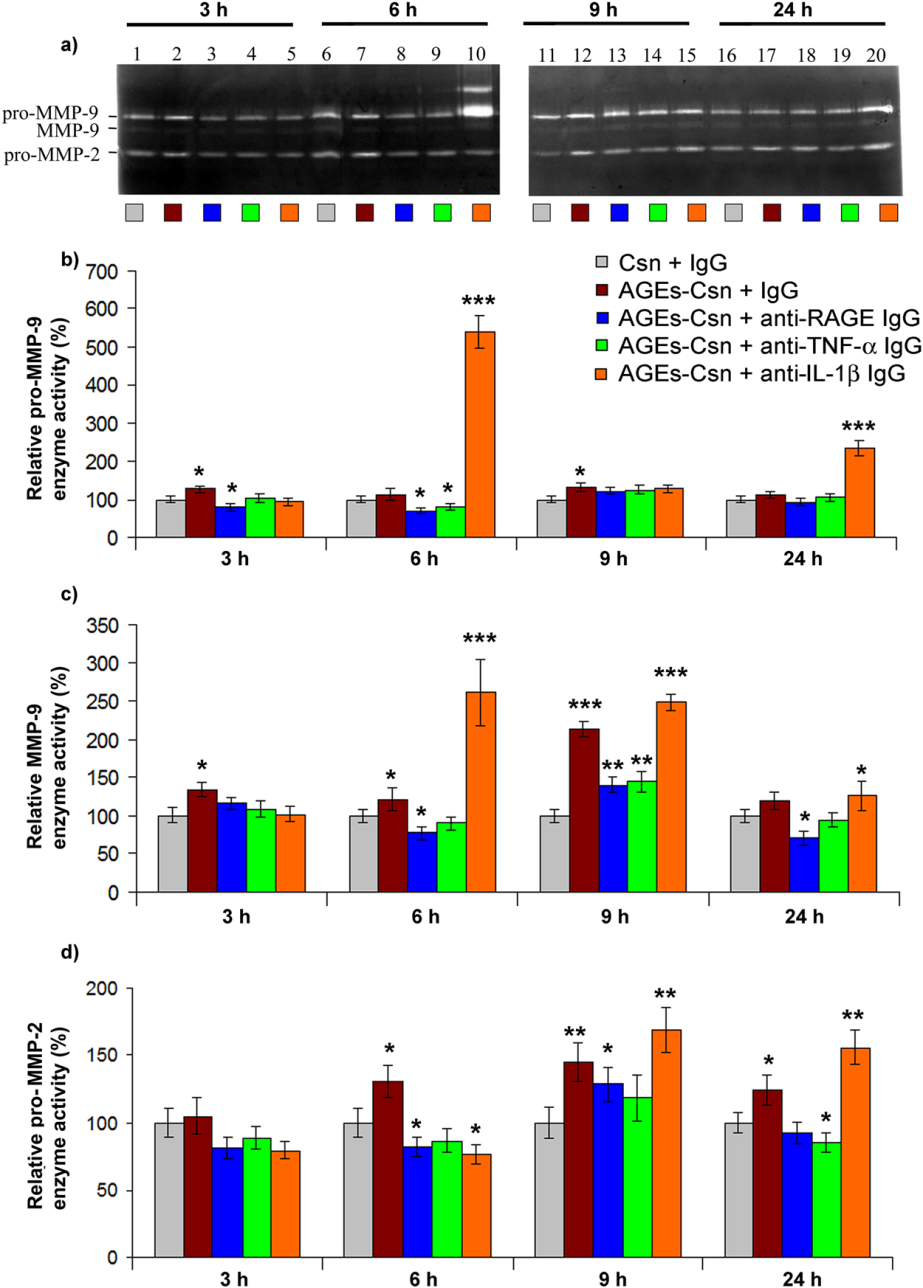
Gelatinolytic activity in C2BBe1 cells exposed to AGEs-Csn. (**a**) Representative zymogram showing pro-MMP-9, MMP-9 and MMP-2 gelatinase activity. The samples at 3 h and 6 h were resolved on a gel that was run in parallel with the samples from 9 h and 24 h. The gel images were cropped, and full-length zymograms are available in Supplementary Fig. 2. In line 10, the NGAL-MMP-9 complex is visible above the band corresponding to pro-MMP-9. (**b**) Densitometry analysis of lysis bands corresponding to pro-MMP-9. (**c**) Densitometry analysis of lysis bands corresponding to MMP-9. (**d**) Densitometry analysis of lysis bands corresponding to MMP-2. Densitometry data represent the means of three experiments and are shown relative to the control from the appropriate time-point. Statistically significant changes (Student’s t-test, Quattro Pro X3 software, Version 13.0.0.406, Mountain View, CA, USA) is noted as *p ≤ 0.05; **p ≤ 0.01; and ***p ≤ 0.001.

The profiles of SOD and CAT activities following AGEs-Csn and non-immunogenic IgG co-exposure created an antioxidative deficit that allowed the intracellular accumulation of H_2_O_2_, which increased by a maximum of 129% at the 6-h time-point (Fig. 2c). At this time-point, the highest level of H_2_O_2_ was also recorded for the AGEs-Csn and anti-IL-1β treatment, which was expected given the high SOD and low CAT activities. The role of AGEs-induced signalling involving RAGE and TNF-α leading to oxidative stress was supported, as both blocking antibody co-treatments effectively prevented major intracellular H_2_O_2_ increases and even managed to reduce the levels to below basal levels after 24 h (Fig. 2c). High SOD (particularly MnSOD) and reduced CAT activity have been described in cancer cell lines^24^, while increased H_2_O_2_ was linked to the development of metastasis and resistance to pro-apoptotic stimuli^31,32^. Increased H_2_O_2_ was suggested to support the expression of MMPs, a family of proteinases that are critically involved in the metastatic process^33^. Our results revealed both the increased protein expression of several MMPs (see Supplementary Table 1) and the activation of MMP-2 and MMP-9 (Fig. 3), particularly in the experimental conditions that also induced increased SOD activity (AGEs-Csn co-treatment with non-immune IgG or anti-IL-1β). The link between moderate levels of H_2_O_2_ and the initiation of cell signalling that favours tumour development was recently discussed, as the phosphorylation of mTOR (Ser2448 and Ser2481) and PKB/Akt phosphorylation (Thr308 and Ser473) were shown to be induced by H_2_O_2_. Interestingly, the former was shown to be induced at concentrations lower than those required for inducing PKB/Akt phosphorylation^34^.

**Figure 4:**
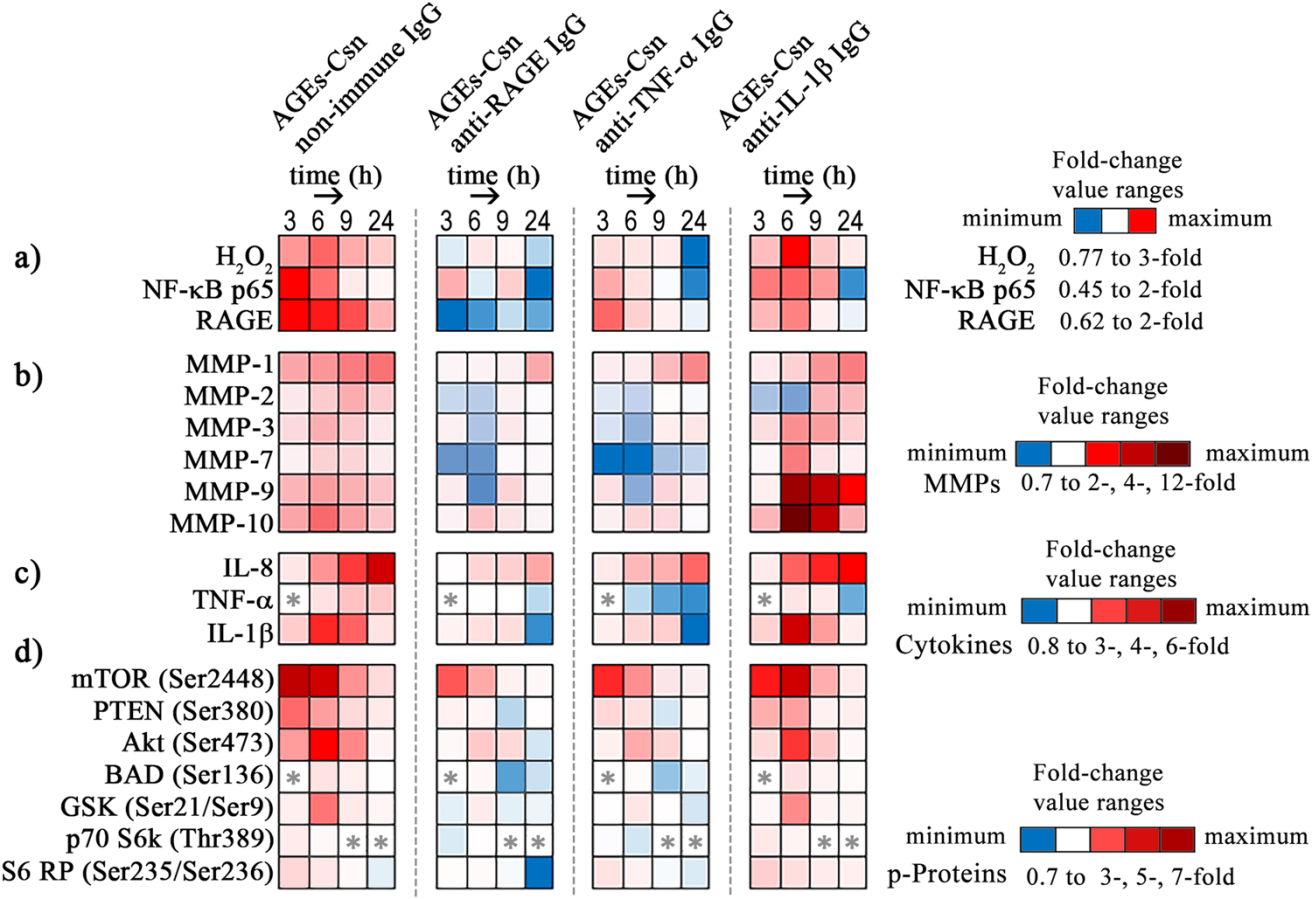
Heat map representing the variation of selected parameters shown as the fold change relative to Csn + non-immune IgG-exposed controls at the appropriate time-point. (**a**) Fold changes were calculated using the data presented in Figs. 2 and 5c,f. (**b**) Fold changes were calculated using the data presented in Supplementary Table 1. (**c**) Fold changes were calculated using the data presented in Supplementary Table 2. (**d**) Fold changes were calculated using the data presented in Supplementary Table 3. Red tones represent relative increases, with the darker reds corresponding to the highest relative fold increases. Blue tones represent decreases relative to the controls, and darker blues correspond to larger fold decreases. White cells correspond to values that are similar to the controls. White cells with an asterisk symbol (*) designate samples in which the assessed analyte was *not detected*.

The highest levels of H_2_O_2_ (1.1 mM) were detected at the 6-h time-point with AGEs-Csn and non-immune IgG co-treatment; additionally, with AGEs-Csn and anti-IL-1β, the intracellular H_2_O_2_ was 1.5 mM. These peaks corresponded to the highest levels of phosphorylated Akt (Ser473) (Fig. 4a–d). Although CAT is typically induced by its substrate, data from the early literature indicated that in Caco-2 cells, CAT activity correlated well with its gene expression levels, in contrast to SOD, whose activity was not shown to be regulated at the transcriptional level^35^. Reports seem to agree that the inflammatory induction of NF-κB can only be accomplished when Nrf2 (a transcription factor that controls the expression of both the SOD and CAT genes) is suppressed, downregulating the radical scavenger function^36^. Such a scenario could occur in our experimental conditions, as we observed an upregulation of NF-κB p65 and intracellular H_2_O_2_ levels in AGEs-Csn and non-immune IgG- or anti-IL-1β-exposed cells after 3 h and 6 h (Figs. 2c and 4a).

Our results revealed that in the AGEs-Csn and non-immune IgG or anti-IL-1β co-treatments, at the 6-h time-point, Akt phosphorylation significantly increased (see Fig. 4d and Supplementary Table 2). This increase in Akt phosphorylation may have contributed to the reduction in CAT activity in an increased H_2_O_2_ background (Fig. 2b,c), as research has already shown for a different cell model^37^. Interestingly, the highest CAT activity that we reported at 3 h and 6 h was in the AGEs-Csn and anti-TNF-α co-treatment condition (Fig. 2b), which may also have been due to the diminished inhibitory effect of TNF-α on CAT expression, as previous research indicated in Caco-2 cells^38^. Along these lines, in the case of AGEs-Csn and non-immune IgG co-treatment, TNF-α gradually accumulated in the culture medium and reached 55.9 pg/µg protein at the 24-h time-point, an amount that could have also contributed to the inhibition of CAT (Supplementary Table 2).

The total antioxidative capacity of C2BBe1 cells was evaluated based on the cell lysate ability to reduce ferric ion, which is a measure of both antioxidative enzyme function as well as the level of reduced compounds with antioxidative properties. With the exception of the small increase in antioxidative capacity at the 3-h time-point, in the conditions of AGEs-Csn and non-immune IgG co-treatment, for the remaining experimental period, the antioxidative capacity was consistently diminished (Fig. 2d). AGEs-Csn and anti-RAGE, as well as AGEs-Csn and anti-TNF-α co-treatments, were very effective not only in preventing the decrease in cell antioxidant capacity at all time-points analysed but also in managing to significantly increase the antioxidant capacity, which peaked at the 9-h time-point by 76% and 139%, respectively. Again, AGEs-Csn and anti-IL-1β antibody co-treatment induced a particular antioxidative capacity profile, which reached a minimum at the 6-h time-point (67% of the control level), rose and peaked at the 9-h time-point (169% relative to the control) and remained significantly elevated until the last time-point that was analysed (Fig. 2d). These results confirmed the presence of an oxidative imbalance that resulted from AGEs-Csn and non-immune IgG treatment, as at the 24-h time-point protein carbonyls were significantly increased and were 33% higher than the control level (Fig. 2e,f). Moreover, increased levels of protein carbonyl groups were also detected with AGEs-Csn and anti-IL-1β antibody co-treatment as early as after 6 h of exposure (Fig. 2e,f). These increases in carbonylated protein were concomitant with decreases in the antioxidative capacity and CAT activity and with increase in H_2_O_2_ levels. Subsequent to the increase at 6 h with AGEs-Csn and anti-IL-1β exposure, carbonylated protein levels returned to the control values. This restoration was probably aided by the notable increases in the MMP-9 and MMP-10 protein expression levels (up to 12-fold at the 6-h time-point; Fig. 4b and Supplementary Table 1) as well as the activity of the 120-kDa neutrophil gelatinase-associated lipocalin (NGAL)-MMP-9 protein complex, pro-MMP-9 and MMP-9 (Fig. 3a–c).

Literature reports revealed that IL-1β–induced deleterious effects and MMP-9 expression were inversely related. The mechanism responsible was reported to involve IL-1β degradation by MMP activity. MMP activity was revealed to play an important role in regulating cytokine function as well as the expression of cytokine receptors. For example, MMP-2 and MMP-9 can degrade IL-1β into biologically inactive fragments but can also activate pro-IL-1β^39^. Additionally, MMP-9 can cleave and inactivate the type II receptor of IL-1β^40^, while MMP-3 was shown to modulate IL-1β function^40,41^. Our results indicated that MMP-3 protein expression was induced by AGEs and anti-IL-1β treatment at the 6-h time-point. Subsequently, MMP-3 expression slightly diminished towards the end of the experimental period, although it remained significantly higher than the control. Similar increases were noted for MMP-7, MMP-9 and MMP-10, and the last two exhibited surprisingly high fold increases of over 11- and 12-fold, respectively, compared to the control (see Fig. 4b and Supplementary Table 1). MMP-3 and MMP-10 have been shown to participate in MMP-9 proteolytic activation^42^; additionally, MMP-7 was recently reported to play a role^43^. Our data show that their protein expression had similar trends to the increases in MMP-9 activity, specifically with AGEs-Csn and anti-IL-1β antibody co-treatment (see Fig. 4b and Supplementary Table 1), which supports the idea that these proteins are functionally linked, as previous research has suggested.

The fact that MMP-7 was shown to mediate tumour growth and metastasis^44^ and that we found that its expression increased following AGEs exposure further supports the role of AGEs in cancer development. As AGEs and anti-IL-1β antibody co-treatment increased the protein expression of MMP-3, MMP-7, MMP-9 and MMP-10 even more than the AGEs-Csn and non-immune IgG conditions (Fig. 4b and Supplementary Table 1), we considered the possibility that at least some metalloproteinases were augmented as a specific response to the blocking antibody.

**Figure 5:**
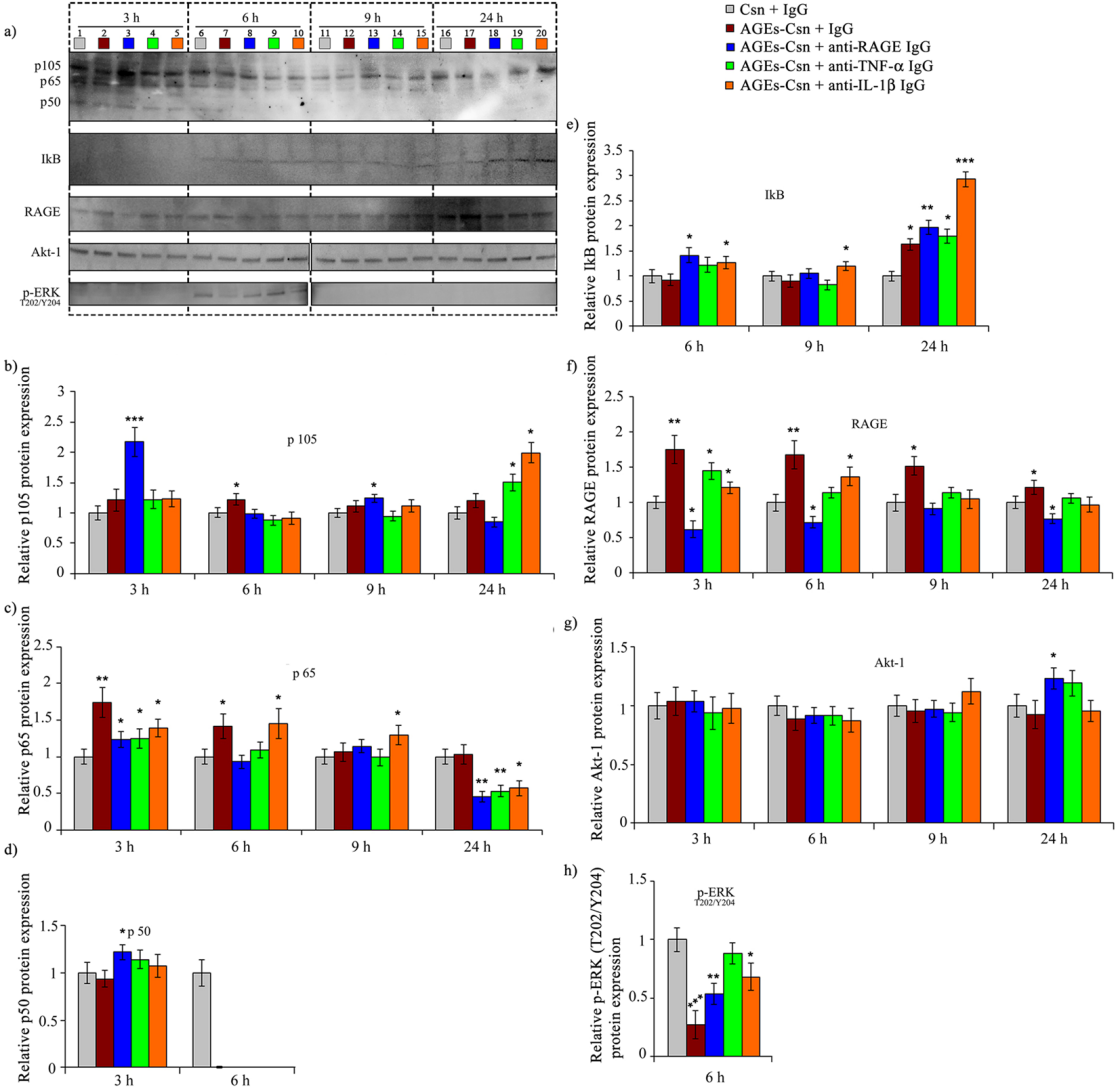
Protein expression profiles of C2BBe1 cells exposed to 200 µg/mL AGEs-Csn and blocking antibodies. (**a**) Representative immunoblots; the time-point is indicated on the top, and the various treatments are colour-coded. Full-length blots are available in Supplementary Figs. 3–5. Samples in lines 1 to 20 were resolved together, with the exception of Akt-1 and p-ERK, for which two gels (indicated by the white spaces left between lines 10 and 11) were used. The limits of the cropped blots are indicated by uninterrupted black edges. (**b**) Densitometry analysis of p105 protein expression. (**c**) Densitometry analysis of p65 protein expression. (**d**) Densitometry analysis of p50 protein expression. (**e**) Densitometry analysis of IkB protein expression. (**f**) Densitometry analysis of RAGE protein expression. (**g**) Densitometry analysis of Akt-1 protein expression. (**h**) Densitometry analysis of p-ERK protein expression. Densitometry analysis data represent the relative fold change in the mean value ± the standard deviation and are reported relative to the control at the corresponding time-point. Densitometry data represent the means of three experiments. Statistical analysis was performed on data groups that were used to calculate the means (Quattro Pro X3 software, Version 13.0.0.406, Mountain View, CA, USA), and significant changes (Student’s t-test) were noted as *p ≤ 0.05; **p ≤ 0.01; and ***p ≤ 0.001.

At the 6-h time-point, AGEs and anti-IL-1β also resulted in the formation of an NGAL-MMP-9 complex (Fig. 3a) that can promote pro-survival and metastatic outcomes and stabilize metalloproteinases, protecting them from degradation^45^; this complex probably further enhanced the gelatinolytic activity at the 6-h time-point (Fig. 3a–c). Although NGAL can be induced by the transcription factor NF-κB, the protein expression of NF-κB protein p65 upon AGEs and anti-IL-1β co-treatment was very similar to the expression found in the AGEs and non-immune IgG conditions, which did not contain the NGAL complex (Figs. 4 and 5a,c). This observation may indicate that other mechanisms may contribute to increased NGAL and MMP-9, such as IL-1β^46^.

Interestingly, IL-1β was shown to activate NF-κB and to increase tight junction permeability in Caco-2 cells^47^. This cell type was shown to be IL-1β hyposensitive, probably as an adaptation rooted in their physiological function, which involves continuous contact with antigens^47^; thus, cells exposed to IL-1β do not undergo apoptosis and may undergo the loosening of the extracellular matrix at the stromal cell and tumour boundary as a result, increasing intestinal cancer invasiveness.

Interestingly, MMP-1 expression was not additionally increased by AGEs-Csn and anti-IL-1β co-treatment than by AGEs-Csn and non-immune IgG co-treatment (see Fig. 4b and Supplementary Table 1), in contrast to the expression profiles of MMP-3, MMP-7, MMP-9 and MMP-10. This observation supports the important role of AGEs and RAGE in MMP-1 upregulation in cancer progression, as the increased expression of MMP-1 (as well as of other MMPs, such as MMP-2, -7, -9 and -13) contributed to poor prognosis in colorectal cancer patients^48^. Moreover, the protein expression of MMP-2 and the gelatinolytic activity of pro-MMP-2 were significantly increased at the 6-h time-point with AGEs-Csn and non-immune IgG treatment and after 9 h in the AGEs-Csn and anti-IL-1β co-treatment condition, subsequent to the large increase in MMP-9 (see Fig. 3 and Supplementary Table 1), possibly as a result of the IL-1β expression peak and pro-inflammatory signalling (see Supplementary Table 2). The increased MMP-2 activity may have potentially behaved as a negative regulatory mechanism by proteolytically cleaving the IL-1β cytokine, as recently described^39^, acting in concert with MMP-9, which targets the cytokine receptor^40^.

The anti-TNF-α and anti-RAGE blocking antibody treatments effectively prevented and diminished the AGE-induced augmentation of MMP-7 (Fig. 4b and Supplementary Table 1). The strong inhibition of MMP-7 protein expression in response to TNF-α antibody blockade also coincided with decreases in extracellular TNF-α expression (Fig. 4c and Supplementary Table 2). Importantly, MMP-7 was shown to be involved in tumour cell survival, growth and invasive potential^49^.

C2BBe1 enterocytes responded to AGEs-Csn and non-immune IgG treatment with a gradual accumulation of IL-8 in the cell culture media, which reached a maximal level of 354 pg IL-8/µg protein at the 24-h time-point, which represented a 6.3-fold increase compared to the control. Although IL-8 levels at 24 h with blocking antibodies were lower than those in response to AGEs-Csn and non-immune IgG treatment, the IL-8 levels were still significantly higher than those in the control, indicating that anti-RAGE, anti-TNF-α and anti-IL-1β antibodies do not effectively prevent IL-8 increases (Fig. 4c and Supplementary Table 2). However, AGEs-Csn and anti-IL-1β co-treatment induced higher IL-8 levels than the AGEs-Csn and non-immune IgG treatment at the 6- and 9-h time-points. The literature shows that MMP-9 is a potent regulator of inflammation that is involved in the proteolytic processing of CXCL8/IL-8 and its subsequent activation in a fashion that is similar to MMP-1 activity^40^. As shown in Fig. 4 and in Supplementary Table 1, the protein expression of MMP-1 and the activity level of MMP-9 (Fig. 3c) paralleled the level of IL-8 (see Supplementary Table 2) in both co-treatment with AGEs and non-immune IgG or anti-IL-1β. Epithelial-mesenchymal transition (EMT) is a process in which epithelial cells gain an aggressive mesenchymal phenotype and is a crucial stage in tumour metastasis. IL-8 is fundamentally involved in several stages of EMT^50^, as it perpetuates a pro-inflammatory microenvironment and promotes its own gene expression through a positive feedback loop^51^.

In some cancers, IL-8 was also shown to activate Akt signalling^52,53^. This observation was supported by our data, since Akt was active and phosphorylated at Ser473 during AGEs and non-immune IgG or anti-IL-1β co-treatment as early as after 3 h of exposure (Fig. 4d, Supplementary Table 3). Akt signalling mechanisms include the phosphorylation of GSK3β, which is rendered inactive, thus preventing GSK3β from phosphorylating and inactivating transcription factors involved in EMT^54^. Our results showed that GSK3β was phosphorylated as early as 3 h after AGEs-Csn exposure and reached its peak at the 6-h time-point (Fig. 4d, Supplementary Table 3). Although AGEs-triggered EMT was not addressed in this study, the accumulation of IL-8 and the activation of MMPs indicate that it is highly probable that AGEs could induce EMT.

Nuclear IκBα prevents p65 from binding to the promoter sequences of the *TNFA* and *IL1B* genes; however, nuclear IκBα was shown to be unable to prevent phosphorylated p65 (S536) from attaching to the *CXCL8* gene promoter^55^. The ribosomal protein S6 kinase (p70S6K) can phosphorylate p65 as well as the ribosomal protein S6 (S6RP). Phosphorylated S6RP was most elevated in our experimental conditions at the 3-h time-point after exposure to AGEs and gradually diminished until it reached the control levels at the 9-h time-point (Fig. 4d, Supplementary Table 3). At the 3-h time-point, we also observed the most elevated levels of p65 protein expression (Fig. 5a–c), which, together with the aforementioned data, could explain why the IL-8 cytokine levels continually increased throughout the 24 h of AGEs exposure, escaping the inhibitory effect of IκBα. Conversely, TNF-α and IL-1β levels started to decrease after 6 h, with TNF-α even reaching levels lower than those of the controls at the 24-h time-point with all antibody treatments (Fig. 4c and Supplementary Table 2), and IκBα was simultaneously significantly increased (Fig. 5a–e). Reports have suggested that TNF-α is proteolytically cleaved and activated by MMP-7^40^. Our results indicated that the TNF-α protein levels showed a mildly increasing trend up to the 24-h time-point (Fig. 4c and Supplementary Table 2); this could potentially be related to the large increase in MMP-7 in the culture media of AGEs-exposed cells, regardless of the antibody co-treatments (Fig. 4b and Supplementary Table 1). Moreover, at the 6-h time-point, although the MMP-7 metalloproteinase protein levels were much higher with anti-IL-1β co-treatment than with non-immune IgG co-treatment, the TNF-α levels were very similar, which was most likely due to the IκBα intervention (Fig. 5e).

### AGEs-Csn activate the RAGE-NF-κB and Akt-mTORC2 signalling pathways, strengthening the link between an AGEs-rich diet and colorectal cancer

Following the 3 h of AGEs-Csn and non-immune IgG exposure, the protein expression of both p65 and RAGE increased by approximately 1.8-fold compared to those in the control, which was probably encouraged by the absence of IκB at this time-point. Many factors that lead to IκB phosphorylation and the subsequent proteasomal degradation^55^ may contribute to the absence of IκB at this time-point (Fig. 5a–e). Additionally, at the 3 h time-point, AGEs-Csn and anti-RAGE antibody co-treatment induced a significant increase in the p50 precursor p105 (the increase was 2.2-fold higher than the control and 1.8-fold higher than the AGE-Csn and non-immune IgG conditions) as well as that of p50 (with an increase of 1.2-fold compared to the control and 1.3-fold compared to the AGEs-Csn and non-immune IgG condition) (Fig. 5a–d). Both p105 and p50 can behave as transcriptional repressors due to their C-terminal ankyrin repeats, which bind and stabilize NF-κB dimers in the cytoplasm^56^. Moreover, previous studies have demonstrated that short-lived p50 is a potent repressor of pro-inflammatory gene expression^57^. Thus, p50 could contribute to inhibiting the expression of the pro-inflammatory molecule TNF-α at the 3-h time-point. Notably, TNF-α was detected only after the p50 levels had decreased at the 6-h time-point (Figs. 4c, 5d and Supplementary Table 2). At the 3-h time-point, all antibody co-treatments had an inhibitory effect on p65 and RAGE protein expression; however, antibody co-treatments were unable to reduce p65 and RAGE protein expression to the control levels, with the notable exception of the RAGE protein level with AGEs-Csn and anti-RAGE antibody co-treatment (Fig. 5a–c,f). At the 6-h time-point, p50 expression was detected in only the control cells, and at later time-points, p50 was undetectable (Fig. 5d). Conversely, the expression level of the IκB inhibitor significantly increased with anti-RAGE or anti-IL1β co-exposure with AGEs-Csn (Fig. 5a–e). After 6 h of AGEs-Csn and non-immune IgG exposure, the protein expression of RAGE and p65 was slightly lower than at the 3 h time-point, although the levels of these proteins were significantly higher than those in the controls, suggesting that IκB had a mild inhibitory effect (Fig. 5a,c,e,f). The main mechanism that regulates NF-κB activity is a negative feedback loop involving NF-κB-induced IκB synthesis and its nuclear translocation, followed by NF-κB binding and transport outside of the nucleus^55^. However, at the 6-h time-point, we noted a decrease in p105 expression for all treatments compared with the levels at the 3-h time-point (Fig. 5a,b). Akt activation probably contributed to this decrease (Fig. 4d and Supplementary Table 2), as Akt could have phosphorylated and inactivated GSK-3β, leading to the downstream destabilization of p105. Additionally, pre-phosphorylated p105 may be subjected to additional phosphorylation via IKK, which is also activated via the PI3K-Akt pathway, ultimately leading to p105 degradation^58^. At the 6-h time-point, AGEs-Csn and anti-IL-1β co-treatment did not alter the increased levels of p65 and RAGE protein expression. In AGEs co-treatment with blocking antibodies, a significant increase in IκB expression contributed to a 2-fold reduction in p65 expression and to the return of the RAGE levels to control values at the 24-h time-point (Fig. 5c,e,f).

The protein expression of Akt-1 was not affected by AGEs exposure or blocking antibody co-treatment at any time-point analysed, with the notable exception of AGEs and anti-RAGE co-treatment for 24 h, which induced a mild increase in Akt protein levels (Fig. 5a–g). Conversely, phosphorylated ERK1/2 (p-ERK1/2) had an interesting expression profile, which was uniquely detectable at the 6-h time-point and was inhibited by AGEs and non-immune IgG exposure by 0.27-fold compared to the controls. In the presence of anti-IL-1β antibodies, both p-Erk1 and p-Erk2 were detected (Fig. 5a–h). The expression profile of p-ERK1/2 may encourage its concerted activity with p105 (Fig. 5a,b), which, on top of the NF-κB inhibitory activity that was already discussed, could also inhibit MAPK activation downstream of Toll-like receptors and TNF-α^55^. ERK signalling inhibition was indicated by the decrease in p-ERK1/2 at the 6-h time-point, and its absence at 9 and 24 h (Fig. 5a–h) may support apoptosis inhibition and/or enhanced cell proliferation^59^.

When exposed to AGEs-Csn and non-immune IgG, several signalling proteins involved in the PI3K/Akt pathway were phosphorylated at higher rates than those detected in the control cells. Thus, at the 3-h time-point, the levels of phosphorylated mTOR (S2448), PTEN (S380), Akt (Ser473) and S6RP (Ser235) significantly increased by 6.5-, 3-, 2.33- and 1.5-fold, respectively (Fig. 3d and Supplementary Table 3). p70 S6 kinase was also the most phosphorylated (Thr389) at this time point in the 24 h interval we studied. After 6 h of AGEs exposure, phosphorylated BAD (Ser136) and GSK-3α/β (Ser21/Ser9) levels were 1.39- and 2.88-fold higher than the controls, while phosphorylated Akt (Ser473) levels were 4.4-fold higher (Fig. 4d and Supplementary Table 3); for all of these proteins, the 6-h time-point showed the highest level of phosphorylation that was detected. After 24 h, these proteins returned to the control levels, with the exception of mTOR (S2448), which was still had significantly higher phosphorylation levels. At the 6-h time-point, AGEs-Csn and anti-RAGE antibody co-treatment reduced the phosphorylation levels of BAD, GSK-3α/β and Akt by half compared with the AGEs and non-immune IgG conditions (Fig. 4d and Supplementary Table 3), while anti-IL-1β antibody co-exposure induced either no change or, in the case of mTOR (S2448), a slight increase in phosphorylation compared to AGEs and non-immune IgG.

This study revealed that the exposure of enterocytes to AGEs can induce the phosphorylation of mTOR (S2448) via RAGE, which is essential for the subsequent assembly of the active mTORC2 complex responsible for Akt phosphorylation at Ser473. The fact that Akt was indeed phosphorylated at Ser473 after the first AGEs exposure time-points emphasizes that mTORC2 was active and that Akt kinase capacity was elevated, as previous research has suggested^60^. Moreover, the signalling downstream of Akt could potentially have led to the subsequent phosphorylation of the pro-apoptotic transcription factor FOXO, inhibiting its transcriptional activity and supporting cell proliferation and survival^61^. The fact that p70 6 S kinase phosphorylation peaked at 3 h and decreased afterwards may suggest that after this time-point, the mTORC1 complex became less active, as p70 6 S kinase is phosphorylated downstream of mTORC1^62^; this also demonstrates that there is a rapid and transient activation of mTORC1 in C2BBe1 cells in response to AGEs exposure.

Our results also revealed that IRS1 was not phosphorylated in control cells or as a result of AGEs exposure (data not shown). Unphosphorylated IRS1 and phosphorylated PTEN stabilize the active form of Akt, strengthening the PI3K/Akt signalling pathway, which is associated with the progression of various types of cancers^60,63^. The idea that AGEs stimulate cancer progression is supported by the phosphorylation of BAD at Ser136 at the 6-h time-point due to the activation of Akt in the presence of AGEs and non-immune IgG. In the phosphorylated form, inactive BAD acts as an antiapoptotic factor^64^.

In type II diabetes, Akt phosphorylation at Ser473 by the mTORC2 complex is exacerbated in the absence of phosphorylated p70S6K and IRS1, which represents a negative feedback mechanism^65^. This scenario also seems probable in C2BBe1 enterocytes exposed to AGEs-Csn starting at the 6-h time-point, although further studies are needed. Akt activation also induced GSK-3β phosphorylation, which targets GSK-3β for degradation^54^, preventing it from contributing to MMPs degradation and thus facilitating the extracellular matrix remodelling that is associated with EMT. The fact that the ribosomal protein S6RP was phosphorylated at 3 h of exposure to AGEs-Csn suggests that mTORC1 may become activated even earlier than 3 h, as S6RP is phosphorylated downstream of mTORC1^66^. In cancer patients, the phosphorylated form of S6RP was associated with a poor prognosis^67^. Moreover, the steady increase in MMP-1 protein expression during AGEs exposure, regardless of the treatment, and the activation of MMP-2 and -9, especially at the 24-h time-point, show the invasive potential of this cell type and the ability of these cells to undergo EMT^68^.

In conclusion, AGEs-Csn-exposed C2BBe1 cells with an enterocyte phenotype demonstrated the induction of cell proliferation; pro-inflammatory cytokine IL-8 accumulation; increased MMP-1, -9 and -10 protein expression; MMP-2 and -9 activation; and only low levels of ROS generation, most likely through SOD and CAT misregulation. These changes are significant when considering cancer evolution. Intercellular H_2_O_2_ formation and RAGE upregulation may have transiently triggered pro-inflammatory and survival signalling pathways, mainly through NF-κB (via RAGE) and mTOR. During AGEs-Csn and anti-RAGE or anti-TNF-α antibody co-treatment, cell proliferation was not detected, and the expression of most MMPs remained lower than or slightly higher than in the controls. The expression of the IL-8 cytokine, MMP-1 and -10 and MMP-9 activation were not prevented by any of the co-treatments and was shown to be difficult to restrain. These MMPs are potential drivers of the extracellular matrix degradation and remodelling associated with cancer pathogenesis. The observation of IL-8 upregulation was surprising, particularly in this cell type, which is known for its immune hyporesponsiveness. This cytokine may be considered a second-wave pro-inflammatory stimulus, perpetuating inflammation in response to an initial stimulus. These results may be an important stepping stone in elucidating the link between dietary AGEs intake and the increased risk of aggressive colorectal cancer development.

## Materials and Methods

### AGEs-Csn preparation, characterization and quantification

The procedures used to obtain and characterize AGEs-Csn as well as to quantify the AGEs in AGEs-Csn were previously described^69^. Briefly, 28 mg/mL total Csn was dispersed in 30 mM phosphate buffer saline (PBS) (pH 6.8) in the presence of 116 mM lactose and 55 mM glucose-fructose and was subjected to heat treatment that simulated the steps used in the production of flavoured ultra-high-temperature processing (UHT) milk (70 °C for 30 min and 135 °C for 8 seconds). Subsequently, to simulate the prolonged storage of UHT products, the samples were incubated at 25 °C for 90 days, and the unreacted reducing sugars were removed by filtration using 10-kDa cut-off membrane filter units (Millipore, St. Charles, MO, USA). The total AGEs content of AGEs-Csn and of the control (non-glycated Csn) was 267 and 11 µg/mg protein^69^, respectively. Any bacterial endotoxin contamination was removed from the non-glycated Csn and AGEs-Csn solutions using an Endotoxin Removal Standard Kit (Bio-Rad, Hercules, CA, USA). The success of the purification procedure was confirmed by measuring endotoxin levels in AGEs-Csn and non-glycated Csn solutions using an E-toxate kit (Sigma-Aldrich, St. Louis, MO, USA). Protein concentrations were determined using a Bradford-based assay (Bio-Rad) with BSA as a standard.

### Cell culture and treatment

C2BBe1 cells derived from a clone of the Caco-2 cell line (CRL-2102 from the American Type Culture Collection (ATCC)) were grown in Dulbecco’s Modified Eagle’s Medium (Biowest, Nuaillé, France) supplemented with 0.01 mg/mL human transferrin (PanReac AppliChem ITW Reagents, Darmstadt, Germany), antibiotic-antimycotic solution 1 × (Biowest), 1.5 g/L sodium bicarbonate and 10% foetal bovine serum (Biowest) in a 5% CO_2_ atmosphere at 37 °C. To evaluate the cytotoxicity of AGEs-Csn compounds and to establish the treatment dose, a MTT assay was performed^70^. For this assay, C2BBe1 human enterocyte cells (2.5 × 10^5^/mL) were seeded in complete medium in 12-well Cell-Culture Treated Multidishes (Nunc, Thermo Fisher Scientific, Roskilde, Denmark). The cells were synchronized and exposed to 50, 100 and 200 μg/mL AGEs-Csn or unmodified Csn in serum-free medium for 3, 6, 9 and 24 h. Briefly, after aspirating the media, cells were washed with 1.5 mL of PBS/well, and then 500 μL (1 mg/mL) of MTT solution was added to each well. After 2 h of incubation, the MTT solution was removed, and 500 μL isopropanol was added to dissolve the formazan crystals. The optical density at 595 nm was determined using a microplate reader (680 Microplate Reader, Bio-Rad).

For the AGEs-Csn exposure experiment, cells were harvested at 80% confluence using 0.25% trypsin-EDTA solution (Biowest) and were sub-cultured in 75 cm^2^ flasks (Nunc EasYFlask, Thermo Fisher Scientific). After reaching approximately 60% confluence, C2BBe1 cells were cultured in serum-free growth media for 18 h to induce cell cycle synchronization and were subjected to 3, 6, 9 and 24 h of co-treatment with 200 μg/mL AGEs-Csn and 500 ng/mL anti-RAGE (sc-33662, Santa Cruz Biotehnology, Inc.), anti-TNFα (AHP1212, Bio-Rad) or anti-IL-1β (VMA00633, Bio-Rad) antibodies. The controls for each time-point were treated with 200 μg/mL AGEs-Csn or non-glycated Csn and non-immune mouse IgG (MB Biomedicals, Solon, OH, USA). Cell numbers and cell viability were assessed using a TC20 automated cell counter (Bio-Rad) and a trypan blue exclusion assay. Cell viability (%) was calculated using the formula $({\rm{ \% }})viability=\frac{viable\,unstained\,cells}{viable\,unstained\,cells+dead\,stained\,cells}\times 100$, and cell proliferation is expressed as the number of viable cells/mL.

### Conditioned media preparation

At the end of each treatment interval, culture supernatants were harvested and centrifuged to remove cell debris and were then concentrated using a 3-kDa cut-off membrane filter unit (Millipore). The protein concentration was determined using a Bradford-based assay (Bio-Rad) with BSA as a standard^71^. The conditioned media samples were aliquoted and stored at −80 °C.

### Quantification of inflammatory cytokines and MMPs from concentrated conditioned media

Conditioned media samples with a total protein concentration of 800 μg/mL were used for the detection of secreted IL-2, IL-4, IL-6, IL-8, IL-10, GM-CSF, IFN-γ and TNF-α cytokines using a Bio-Plex Pro Human Cytokine 8-plex panel (Bio-Rad), as previously described^10^. Secreted IL-1β was separately quantified using Bio-Plex Pro Human Cytokine Standard 27-plex, Group I, magnetic beads and detection antibodies for human IL-1β (Bio-Rad). MMP-1, -2, -3, -7, -8, -9, -10, -12 and 13 were assessed using a Bio-Plex Pro Human MMP panel 9-plex (Bio-Rad). The cytokine and MMPs levels were analysed according to the manufacturer’s instructions using a Bio-Plex MAGPIX System and Bio-Plex Manager software version 6.0 (Bio-Rad).

### Gelatine zymography

The gelatinolytic activity of concentrated conditional media samples was assessed after proteins were resolved by sodium dodecyl sulfate-polyacrylamide gel electrophoresis (SDS-PAGE) in 8% polyacrylamide gels containing 10 mg/mL gelatine as a substrate. Samples (25 µg/well total protein) were mixed with Zymogram Sample Buffer (Bio-Rad) and were subjected to SDS-PAGE at 125 V for 95 min. The gels were washed in 1X Zymogram Renaturation Buffer (Bio-Rad) for 30 min at room temperature to remove SDS and to renature the MMPs. Gels were incubated overnight at 37 °C with 1X Zymogram Development Buffer (Bio-Rad) and stained with 0.5% Coomassie blue R-250 in 40% v/v methanol and 10% v/v acetic acid aqueous solution for 30 min at 37 °C. After destaining the gels, the MMPs gelatinase activity was detected using a ChemiDoc MP System and was quantified by densitometry using Image Lab software version 5.2.1 (Bio-Rad).

### Cell lysate preparation

At each experimental interval, the cells were detached using 0.25% trypsin-EDTA, centrifuged and re-suspended in PBS. The cell suspension was sonicated (three times, 30 seconds, on ice). The cell debris were removed by centrifugation at 10000 relative centrifugal force (RCF) for 10 min at 4 °C. Finally, the supernatant was aliquoted and frozen at −80 °C for subsequent biochemical and immunochemical analyses.

### Antioxidant enzyme activity and oxidative stress markers

Total intracellular SOD activity was assessed using a DetectX SOD Colorimetric Activity Kit (Arbor Assays, Ann Arbor, MI, USA) according to the manufacturer’s instructions. The SOD activity in the standard curve was between 4 and 0.0625 U/mL. The intracellular catalase activity was assessed with a colorimetric method using a DetectX CAT Colorimetric Activity Kit (Arbor Assays) with a horseradish peroxidase (HRP) standard between 5 and 0.156 U/mL. The intracellular level of H_2_O_2_ was detected using a colorimetric substrate in the presence of HRP that was provided by a DetectX Hydrogen Peroxide Colorimetric Detection Kit (Arbor Assays). The antioxidant capacity of cell lysates was evaluated using DetectX Ferric Reducing Antioxidant Power (FRAP) (Arbor Assays) according to manufacturer’s instructions.

### Western blot assays

For target protein expression evaluation, whole-cell protein lysates were resolved on Protean TGX Stain Free 4–15% precast gels (Bio-Rad), transferred onto 2-μm PDVF (V3 Western Workflow, Bio-Rad) and the total transferred protein signal was detected and quantified using a ChemiDoc MP System and Image Lab software (version 5.2.1, Bio-Rad). The membranes were blocked using 5% non-fat dry milk in TBST for 2 h at room temperature. A mouse anti-human NF-κB p105 monoclonal antibody (MCA4802Z, 1:1000 dilution factor), mouse anti-human NF-κB p65 monoclonal antibody (sc-8008, 1:200 dilution factor), mouse anti-human NF-κB p50 monoclonal antibody (VMA00673, 1:1000 dilution factor), mouse anti-human IκB monoclonal antibody (MCA4948Z, 1:200 dilution factor), mouse anti-human ERK (pThr202/pTyr204) monoclonal antibody (MCA5990GA, 1:1000 dilution factor), and mouse anti-human Akt-1 monoclonal antibody (MCA4779Z, 1:200 dilution factor) were used. HRP-conjugated secondary antibodies (STAR120 and STAR133, 1:1000 dilution factor, Bio-Rad) were used. To immunostain the membranes, they were incubated with the primary antibodies for 2 h and with the secondary antibodies for 1 h at room temperature. Blots were developed using Clarity Western ECL Substrate (Bio-Rad), and the chemiluminescence signal was detected using a ChemiDoc MP System. The target protein expression was quantified using Image Lab software version 5.2.1 (Bio-Rad) and was normalized to the total protein transferred to the membrane (each protein band was normalized against the total protein transferred in the corresponding lane).

Cellular carbonylated protein detection was performed using an OxiSelect Protein Carbonyl Immunoblot Kit (Cell Biolabs, San Diego, CA, USA) with a post-transfer derivatization step for protein-bound carbonyl groups performed by using a solution of dinitrophenylhydrazine (DNPH). The adducts that formed were recognized by the primary rabbit anti-DNPH (diluted 1:1000) and an HRP-conjugated secondary antibody, anti-rabbit IgG (diluted 1:1000).

### Evaluation of the phosphorylation of proteins in the AKT signalling pathway

The phosphorylation levels of Akt (Ser473), BAD (Ser136), GSK-3α/β (Ser21/Ser9), IRS-1 (Ser636/Ser639), mTOR (Ser2248), p70 S6 kinase (Thr389), PTEN (Ser380) and S6 ribosomal protein (Ser235/Ser236) were assessed in whole cell lysates (total protein concentration of 800 μg/mL) using a Bio-Plex Pro cell signaling Akt 8-plex panel (Bio-Rad) according to the manufacturer’s instructions and the Bio-Plex MAGPIX System with Bio-Plex Manager software version 6.0 (Bio-Rad).

### Statistical analysis

All data are presented as the mean ± the standard deviation. All tests were performed in at least triplicate. Statistical significance was calculated using a Student’s t-test (two-tailed distribution with two-sample unequal variance).

## Supplementary information

Supplementary information.
