# Supplementary material for: Dietary AGEs involvement in colonic inflammation and cancer: insights from an in vitro enterocyte model
Source: Sci Rep. 2020 Feb 17;10:2754. doi: 10.1038/s41598-020-59623-x (PMC7026081; doi:10.1038/s41598-020-59623-x)
Supplement: Supplementary file 1 — Supplementary information. [file 41598_2020_59623_MOESM1_ESM.doc]

**Dietary AGEs involvement in colonic inflammation and cancer: insights from an *in vitro* enterocyte model**

Ovidiu I. Geicu^1, 2^, Loredana Stanca^2^, Sorina NicoletaVoicu^1^, Anca Dinischiotu^1^, Liviu Bilteanu^2^ Andreea I. Serban*^1, 2^, Valentin Calu^3^

^1^Department of Biochemistry and Molecular Biology, Faculty of Biology, University of Bucharest, 91-95 Splaiul Independentei, 050095, Bucharest, Romania

^2^Department of Preclinical Sciences, Faculty of Veterinary Medicine, University of Agronomic Sciences and Veterinary Medicine of Bucharest, 105 Splaiul Independentei, 050097, Bucharest, Romania

^3^Department of General Surgery, University of Medicine and Pharmacy "Carol Davila" Bucharest, 8 Bulevardul Eroii Sanitari, 050474, Bucharest, Romania

*Correspondence should be addressed to A.I.S. (email: irensro@yahoo.com; andreeaserban@fmvb.ro).

**Supplementary Information**

**Supplementary Table 1.** The absolute protein levels (pg/mL) of MMP-1, -2, -3, -7, -9 and MMP-10 (conditioned media protein concentration was 800 µg/mL).

| MMPs | Treatments | Absolute values (pg/mL)  Exposure duration | | | |
| --- | --- | --- | --- | --- | --- |
|  |  | 3 h | 6 h | 9 h | 24 h |
| MMP-1 | Csn + IgG | 65.3±4.1 | 72.1±6.5 | 82.2±8.1 | 91.3±8.7 |
|  | AGEs-Csn + non-immune IgG | 107.4±9.2* | 126.8±9.9* | 160.1±11.2* | 185.1±14.5* |
|  | AGEs-Csn + anti-RAGE IgG | 69.5±5.2 | 77.9±7.1 | 91.1±8.2 | 147.8±12.1* |
|  | AGEs-Csn + anti-TNF-α IgG | 73.3±6.8 | 82.1±7.4 | 122.3±11.3* | 170.7±15.7* |
|  | AGEs-Csn + anti-IL-1β IgG | 77.9±8.1 | 95.4±9.2* | 145.3±12.1* | 177.9±15.9* |
| MMP-2 | Csn + IgG | 112.2±8.9 | 136.4±11.5 | 151.6±14.1 | 175.1±15.9 |
|  | AGEs-Csn + non-immune IgG | 129.2±11.2 | 187.0±14.8* | 243.1±19.7* | 236.3±21.4* |
|  | AGEs-Csn + anti-RAGE IgG | 101.6±9.3 | 119.2±10.7 | 164.7±15.8 | 178.7±16.1 |
|  | AGEs-Csn + anti-TNF-α IgG | 107.4±9.2 | 126.2±11.7 | 153.8±15.1 | 173.2±16.9 |
|  | AGEs-Csn + anti-IL-1β IgG | 94.3±8.4 | 101.9±8.9* | 232.2±20.7* | 263.1±21.8* |
| MMP-3 | Csn + IgG | 24.2±2.1 | 34.3±3.1 | 37.5±3.3 | 41.2±4.1 |
|  | AGEs-Csn + non-immune IgG | 30.5±2.9* | 54.1±4.8* | 52.3±4.6* | 47.3±4.3 |
|  | AGEs-Csn + anti-RAGE IgG | 26.3±2.5 | 29.3±2.7 | 43.5±4.1 | 42.5±3.9 |
|  | AGEs-Csn + anti-TNF-α IgG | 23.3±2.2 | 27.6±2.6 | 39.7±3.7 | 44.4±4.2 |
|  | AGEs-Csn + anti-IL-1β IgG | 29.9±2.1* | 62.0±5.7* | 64.1±5.9* | 58.1±5.1* |
| MMP-7 | Csn + IgG | 187.2±17.1 | 203.0±19.6 | 210.0±18.5 | 627.4±54.1 |
|  | AGEs-Csn + non-immune IgG | 204.4±19.5 | 267.9±21.6* | 272.3±23.6* | 710.5±67.4 |
|  | AGEs-Csn + anti-RAGE IgG | 135.0±11.1* | 145.4±13.4* | 215.6±18.2 | 649.8±60.2 |
|  | AGEs-Csn + anti-TNF-α IgG | 117.2±10.5* | 124.0±11.6* | 175.8±15.3 | 556.0±48.6 |
|  | AGEs-Csn + anti-IL-1β IgG | 197.1±17.5 | 397.1±31.5* | 251.3±23.5 | 696.1±60.2 |
| MMP-9 | Csn + IgG | 49.6±4.4 | 58.4±5.6 | 63.1±5.7 | 91.4±8.9 |
|  | AGEs-Csn + non-immune IgG | 76.1±6.1* | 99.7±9.2* | 98.7±8.1* | 128.7±10.1* |
|  | AGEs-Csn + anti-RAGE IgG | 56.1±5.4 | 40.4±3.9* | 82.4±7.1* | 96.1±9.1 |
|  | AGEs-Csn + anti-TNF-α IgG | 60.1±5.9 | 45.5±4.1* | 81.5±7.3* | 102.3±9.5 |
|  | AGEs-Csn + anti-IL-1β IgG | 52.4±4.9 | 658.9±57.1*** | 223.9±20.1** | 174.9±16.4* |
| MMP-10 | Csn + IgG | 20.2±1.7 | 25.8±2.1 | 33.8±2.8 | 159.4±13.1 |
|  | AGEs-Csn + non-immune IgG | 33.2±2.6* | 53.8±4.7* | 56.6±5.1* | 214.9±20.1* |
|  | AGEs-Csn + anti-RAGE IgG | 21.9±1.9 | 36.9±2.6* | 40.4±3.8 | 170.0±16.7 |
|  | AGEs-Csn + anti-TNF-α IgG | 22.2±1.8 | 33.5±2.5* | 42.7±3.7* | 162.2±16.1 |
|  | AGEs-Csn + anti-IL-1β IgG | 30.6±2.4* | 314.4±30.1*** | 131.9±11.7** | 247.1±23.2* |

All experiments were performed in triplicate and data are shown as the mean of the absolute values ± the standard deviation (n = 3). Statistical significance as calculated with Student t-test was noted: * p < 0.05, ** p < 0.01, *** p < 0.001.

**Supplementary Table 2.** The absolute cytokine levels (pg /µg total protein) of IL-8, TNF-α and IL-1β in the conditioned media.

| Cytokine | Treatments | Absolute values (pg/µg total protein) | | | |
| --- | --- | --- | --- | --- | --- |
|  |  | Exposure duration | | | |
|  |  | 3 h | 6 h | 9 h | 24 h |
| IL-8 | Csn + non-immune IgG | 37.2±2.9 | 35.3±3.4 | 42.8±3.8 | 56.5±5.4 |
|  | AGEs-Csn + IgG | 50.2±4.6* | 88.5±7.4** | 161.0±15.9** | 354.0±32.6*** |
|  | AGEs-Csn + anti-RAGE IgG | 38.7±3.4 | 56.8±4.9* | 71.9±6.4* | 127.0±10.4* |
|  | AGEs-Csn + anti-TNF-α IgG | 48.6±4.1* | 70.5±6.6* | 91.1±8.8** | 177.0±16.8** |
|  | AGEs-Csn + anti-IL-1β IgG | 48.2±4.4* | 114.0±10.2** | 174.5±16.6*** | 258.2±24.2*** |
| TNF-α | Csn + non-immune IgG | ND | 7.6±0.64 | 8.8±0.75 | 31.8±2.8 |
|  | AGEs-Csn + IgG | ND | 10.8±1.04* | 16.5±1.4* | 55.9±4.4* |
|  | AGEs-Csn + anti-RAGE IgG | ND | 7.9±0.72 | 9.1±0.84 | 30.5±2.8 |
|  | AGEs-Csn + anti-TNF-α IgG | ND | 7.3±0.51 | 7.8±0.64 | 26.9±2.4 |
|  | AGEs-Csn + anti-IL-1β IgG | ND | 10.5±0.9* | 11.5±1.04* | 28.3±2.7 |
| IL-1β | Csn + non-immune IgG | 5.4±0.48 | 7.3±0.68 | 8.5±0.77 | 13.1±1.35 |
|  | AGEs-Csn + IgG | 9.3±0.68* | 29.6±2.72** | 27.3±2.43** | 18.2±1.72* |
|  | AGEs-Csn + anti-RAGE IgG | 6.4±0.49 | 10.7±0.94* | 12.7±1.12* | 11.2±1.04 |
|  | AGEs-Csn + anti-TNF-α IgG | 6.8±0.62* | 11.6±0.92* | 14.5±1.14* | 10.6±1.11 |
|  | AGEs-Csn + anti-IL-1β IgG | 8.9±0.80* | 45.3±4.02** | 20.3±1.94** | 16.4±1.42* |

All experiments were performed in triplicate and data are shown as the mean of the absolute values ± the standard deviation (n = 3). ND – not detected. Statistical significance as calculated with Student t-test was noted: * p < 0.05, ** p < 0.01, *** p < 0.001.

**Supplementary Table 3.** The relative levels of phosphorylated proteins involved in Akt signaling pathway.

| **Protein and phosphorylation site** | **Treatments** | **Relative change vs. control ± standard deviation** | | | |
| --- | --- | --- | --- | --- | --- |
|  |  | **Treatment duration** | | | |
|  |  | **3 h** | **6 h** | **9 h** | **24 h** |
| **mTOR (Ser2448)** | Csn + IgG | 1.00±0.17 | 1.00±0.21 | 1.00±0.19 | 1.00±0.22 |
|  | AGEs-Csn + IgG | 6.51±0.37*** | 4.61±0.55*** | 2.46±0.36** | 1.48±0.17* |
|  | AGEs-Csn + anti-RAGE IgG | 3.28±0.33** | 2.16±0.17** | 1.19±0.18 | 1.12±0.12 |
|  | AGEs-Csn + anti-TNF-α IgG | 3.90±0.27** | 2.53±0.22** | 1.62±0.17* | 1.37±0.21 |
|  | AGEs-Csn + anti-IL-1β IgG | 4.12±0.45*** | 4.89±0.38*** | 2.11±0.29** | 1.28±0.18 |
| **PTEN (Ser380)** | Csn + IgG | 1.00±0.14 | 1.00±0.16 | 1.00±0.11 | 1.00±0.19 |
|  | AGEs-Csn + IgG | 3.02±0.29*** | 2.31±0.17** | 1.51±0.22* | 1.29±0.11 |
|  | AGEs-Csn + anti-RAGE IgG | 1.19±0.11 | 1.14±0.16 | 0.92±0.17 | 1.04±0.09 |
|  | AGEs-Csn + anti-TNF-α IgG | 1.55±0.16* | 1.43±0.19* | 0.95±0.17 | 1.07±0.1 |
|  | AGEs-Csn + anti-IL-1β IgG | 2.10±0.27** | 2.30±0.21** | 1.20±0.17 | 1.23±0.17 |
| **AKT (Ser473)** | Csn + IgG | 1.00±0.12 | 1.00±0.23 | 1.00±0.13 | 1.00±0.17 |
|  | AGEs-Csn + IgG | 2.33±0.17** | 4.43±0.35*** | 2.62±0.24** | 1.15±0.10 |
|  | AGEs-Csn + anti-RAGE IgG | 1.10±0.13 | 1.73±0.18* | 1.53±0.17* | 0.95±0.10 |
|  | AGEs-Csn + anti-TNF-α IgG | 1.21±0.15 | 2.13±0.20** | 1.58±0.15* | 1.03±0.09 |
|  | AGEs-Csn + anti-IL-1β IgG | 1.51±0.21* | 3.73±0.27*** | 1.75±0.21* | 1.12±0.11 |
| **BAD (Ser136)** | Csn + IgG | ND | 1.00±0.09 | 1.00±0.10 | 1.00±0.11 |
|  | AGEs-Csn + IgG | ND | 1.39±0.12* | 1.22±0.17 | 1.02±0.09 |
|  | AGEs-Csn + anti-RAGE IgG | ND | 1.11±0.12 | 0.81±0.09* | 0.94±0.09 |
|  | AGEs-Csn + anti-TNF-α IgG | ND | 1.06±0.10 | 0.88±0.08 | 0.97±0.10 |
|  | AGEs-Csn + anti-IL-1β IgG | ND | 1.42±0.13* | 1.17±0.12 | 1.08±0.08 |
| **GSK (Ser21/**  **Ser9)** | Csn + IgG | 1.00±0.13 | 1.00±0.11 | 1.00±0.08 | 1.00±0.17 |
|  | AGEs-Csn + IgG | 1.22±0.12 | 2.88±0.30** | 1.29±0.14* | 1.14±0.12 |
|  | AGEs-Csn + anti-RAGE IgG | 0.97±0.08 | 1.28±0.09* | 0.97±0.10 | 0.98±0.09 |
|  | AGEs-Csn + anti-TNF-α IgG | 1.03±0.10 | 1.34±0.11* | 1.03±0.09 | 0.95±0.19 |
|  | AGEs-Csn + anti-IL-1β IgG | 1.11±0.09 | 2.58±0.21** | 1.16±0.11 | 1.16±0.12 |
| **p70 S6 kinase (Thr389)** | Csn + IgG | 1.00±0.1 | 1.00±0.07 | ND | ND |
|  | AGEs-Csn + IgG | 1.27±0.17 | 1.1±0.3 | ND | ND |
|  | AGEs-Csn + anti-RAGE IgG | 0.96±0.06 | 1.03±0.09 | ND | ND |
|  | AGEs-Csn + anti-TNF-α IgG | 1.04±0.10 | 0.95±0.11 | ND | ND |
|  | AGEs-Csn + anti-IL-1β IgG | 1.31±0.14 | 1.15±0.17 | ND | ND |
| **S6RP**  **(Ser235/**  **Ser236)** | Csn + IgG | 1.00±0.09 | 1.00±0.12 | 1.00±0.08 | 1.00±0.10 |
|  | AGEs-Csn + IgG | 1.54±0.14* | 1.33±0.11* | 1.09±0.12 | 0.97±0.09 |
|  | AGEs-Csn + anti-RAGE IgG | 1.05±0.10 | 1.07±0.09 | 0.99±0.11 | 0.71±0.09* |
|  | AGEs-Csn + anti-TNF-α IgG | 1.39±0.14* | 1.20±0.13 | 0.99±0.09 | 0.96±0.10 |
|  | AGEs-Csn + anti-IL-1β IgG | 1.66±0.17* | 1.45±0.17* | 1.27±0.12* | 1.21±0.12 |

All experiments were performed in triplicate and data are shown as the mean of the absolute values ± the standard deviation (n = 3). ND – not detected. Statistical significance as calculated with Student t-test was noted: * p < 0.05, ** p < 0.01, *** p < 0.001.

| **Supplementary Figures** | | |
| --- | --- | --- |
| 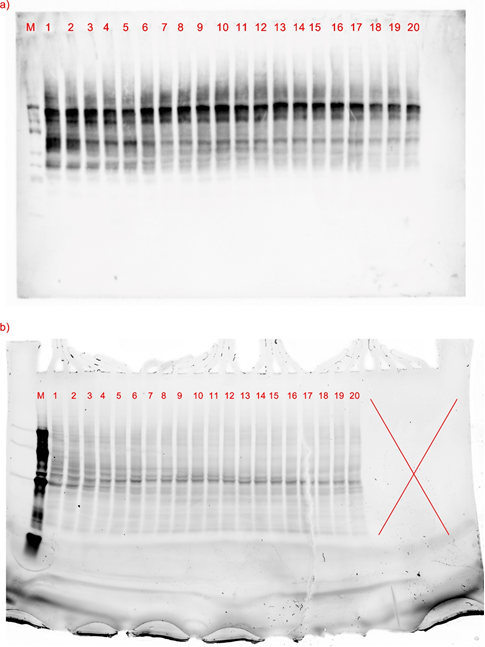 | **Supplementary Figure 1.** a) Original whole western blot corresponding to the image shown in Fig. 2e. b) original whole 4–15% Criterion™ TGX Stain-Free™ Protein Gel (26 well, 15 µl) PAGE gel corresponding to the blot shown in a), with full length lines with resolved protein bands. The image was captured with the ChemiDoc MP System before proceeding to protein transfer to PVDF membrane. For the final figure, Photoshop was used to crop the image shown in a) to remove areas with no signal.  Protein normalization was done using the total proteins loaded after densitometric analysis of the gels with resolved proteins and the results (average of three experiments) are shown in Fig. 2f as relative values. | |
| 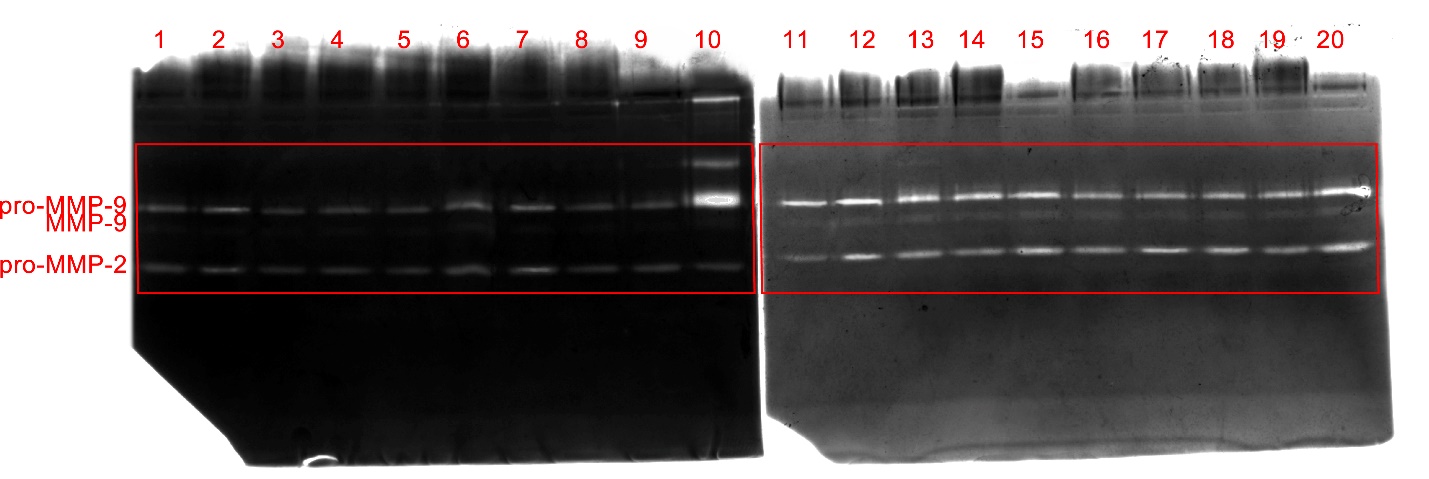 | **Supplementary Figure 2**. Original whole-length zymograms revealing the pro-MMP-9, MMP-9 and pro-MMP-2 gelatinase activities, corresponding to the cropped image shown in Fig. 3a. For the final figure, Photoshop was used to crop the areas indicated by the red rectangles. | |
| 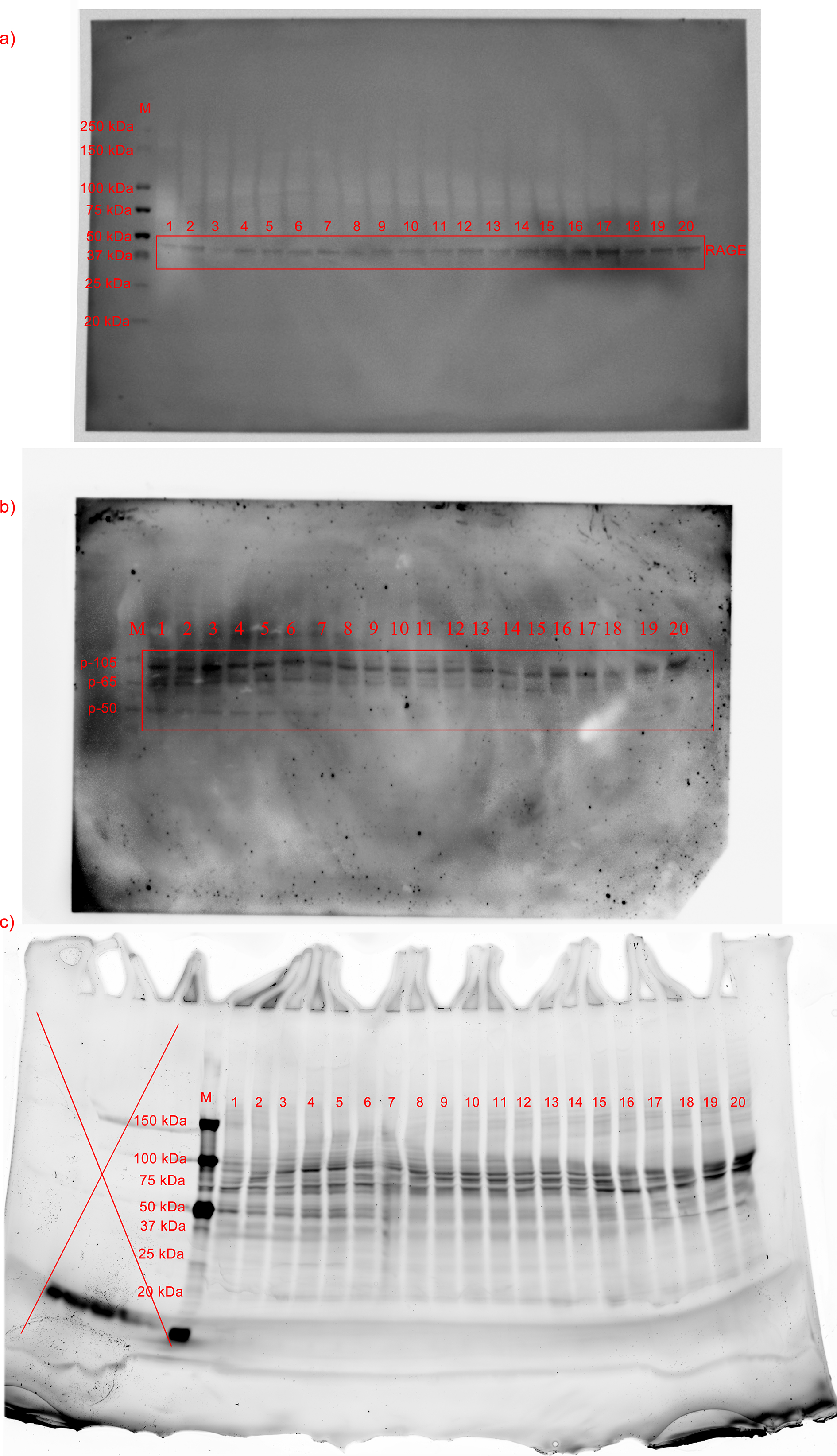 | | **Supplementary Figure 3.** a) Original whole-length western blot revealing the RAGE protein expression, corresponding to the cropped image shown in Fig. 5a. For the final figure 5a, Photoshop was used to crop the area shown in the red rectangle. The blot shown in a) was stripped using Vantage ReView Stripping Buffer and reused for b).  b) Original whole-length western blot revealing the NF-κB p105, p65 and p50 proteins expression, corresponding to the cropped image shown in Fig. 5a. The membrane was incubated alternatively with each primary antibody and washed 3 × 5 min with TBST (Tris-buffered saline, 0.1% Tween 20) wash buffer between each antibody, then incubated with the secondary antibody. For the final figure, Photoshop was used to crop the area shown in the red rectangle.  c) original whole 4–15% Criterion TGX Stain-Free Protein Gel (26 well, 15 µl) showing full length lines with resolved protein bands. The image was captured with the ChemiDoc MP System before proceeding to protein transfer to PVDF membrane.  Protein normalization was done using the total proteins loaded after densitometric analysis of the gels. The gel corresponding to blots shown in a) and b) is shown in c). The results shown in Fig. 5b for NF-κB p105, Fig. 5c for p65, Fig. 5d for p50 and Fig. 5f for RAGE are relative values calculated from the average of three experiments. |
| 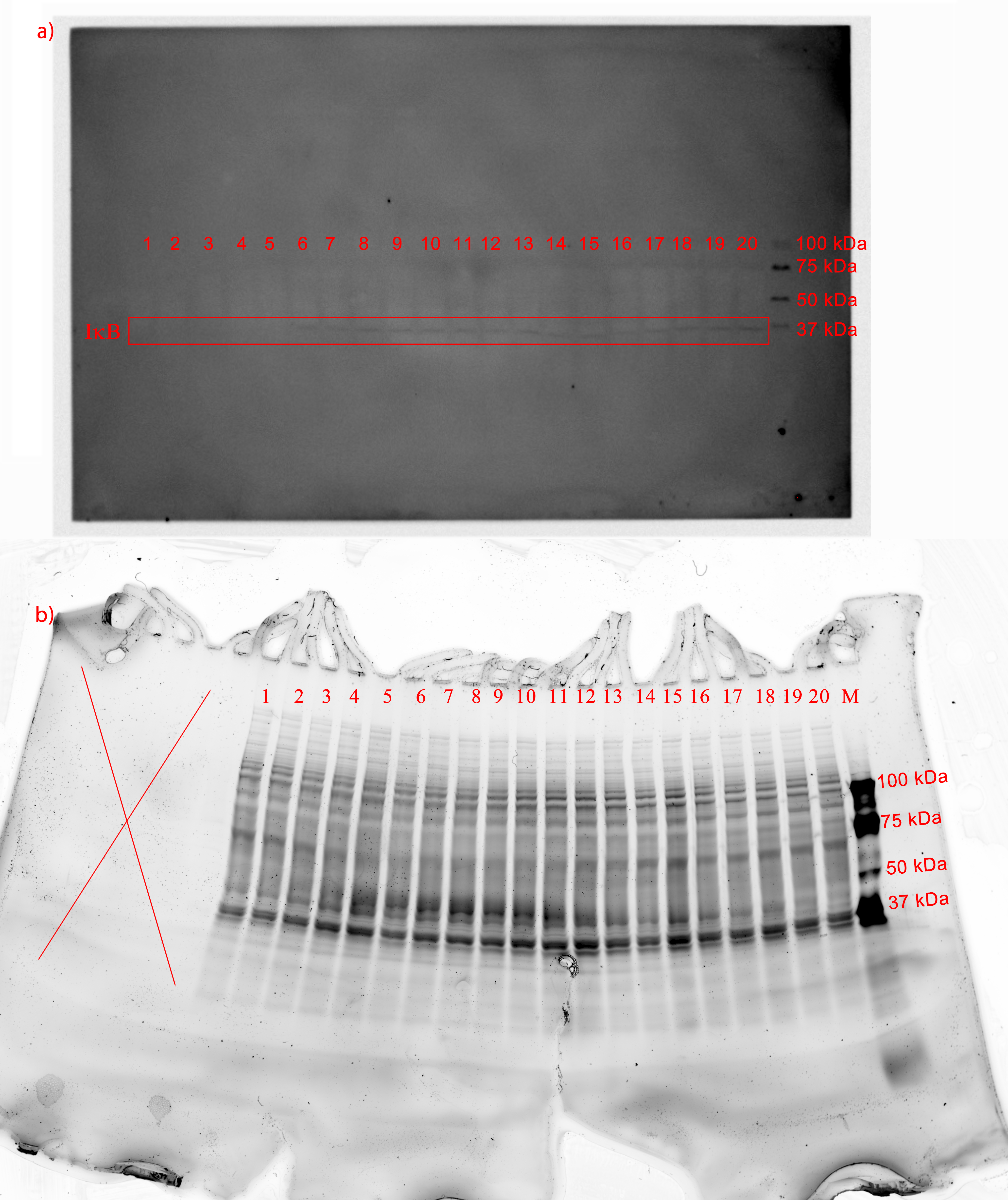 | | **Supplementary Figure 4.** a) Original whole western blot corresponding to the image shown in Fig. 5a representing IκB protein expression. For the final figure 5a, Photoshop was used to crop the horizontal bands as indicated by the red rectangle. The levels were adjusted so that the bands were more apparent. The whole horizontal line of bands was adjusted at the same time. b) original whole 4–15% Criterion TGX Stain-Free Protein Gel (26 well, 15 µl) showing full length lines with resolved protein bands corresponding to the blot shown in a). The image was captured with the ChemiDoc MP System before proceeding to protein transfer to PVDF membrane.  Protein normalization was done using the total proteins loaded after densitometric analysis of the gels with resolved proteins and the results (average of three experiments) are shown in Fig. 5e as relative values. |
| 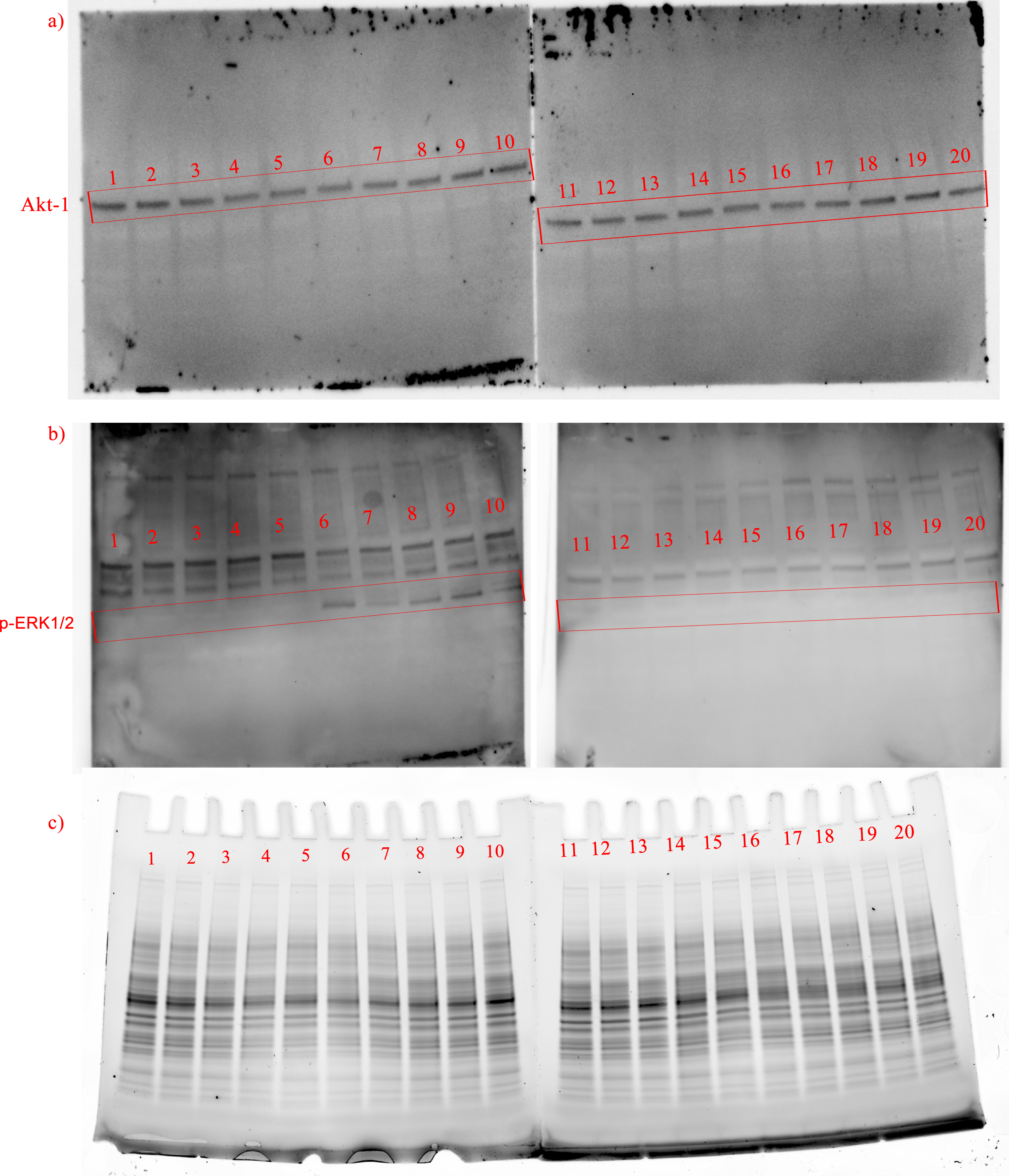 | | **Supplementary Figure 5**. a) Original whole-length western blot revealing the Akt-1 protein expression, corresponding to the cropped image shown in Fig. 5a. For the final figure, Photoshop was used to crop the areas indicated by the red rectangles.  The membranes shown in a) were stripped using Vantage ReView Stripping Buffer and reused for b).  b) Original whole-length western blot revealing the p-ERK1/2 protein expression, corresponding to the cropped image shown in Fig. 5a. For the final figure, Photoshop was used to crop the areas indicated by the red rectangles.  c) original whole gels (10% Mini-PROTEAN TGX Stain-Free Protein Gels, 10 well, 50 µl) showing full length lines with resolved protein bands corresponding to blots shown in a) and b). The image was captured with the ChemiDoc MP System before proceeding to protein transfer to PVDF membrane.  Protein normalization was done using the total proteins loaded after densitometric analysis of the gels and the results shown as relative values (average of three experiments) are shown in Fig. 5g for Akt and Fig. 5h for p-ERK ½. |
